# Implementing an Online Instrument to Measure Nurse Practitioner Workload: A Feasibility Study

**DOI:** 10.1177/21501319251321302

**Published:** 2025-02-19

**Authors:** Kelley Kilpatrick, Véronique Landry, Eric Nguemeleu Tchouaket, André Daigle, Mira Jabbour

**Affiliations:** 1 McGill University, Montréal, QC, Canada; 2 Centre intégré universitaire de santé et de services sociaux (CIUSSS) de l’Est-de-l’Île-de-Montréal—Hôpital Maisonneuve-Rosemont, Montréal, QC, Canada; 3 Université de Montréal, Montréal, QC, Canada; 4 Université de Moncton, Moncton, NB, Canada; 5 Université du Québec en Outaouais, Saint-Jérôme, QC, Canada

**Keywords:** community-based primary care, feasibility study, measurement, mixed methods, nurse practitioners, workload

## Abstract

**Introduction/Objectives:**

Nurse practitioners (NPs) improve access to care in community-based primary care. Determining an appropriate workload for NPs is complex. The number of patients seen by NPs represents an important consideration. We sought to determine the feasibility, acceptability and appropriateness of implementing the online NP workload measurement index (NP-WI).

**Methods:**

Feasibility study supported by the Theoretical Framework of Acceptability, conducted across 3 health regions in Québec, Canada. Data were collected from January to July 2024 using the online NP-WI (*n* = 66), 8-item acceptability questionnaire (*n* = 47), weekly implementation team meetings with NPs and decision-makers (*n* = 11), field notes and interviews (*n* = 13). Data analysis completed using descriptive statistics and content analysis, with data integration using joint displays.

**Results:**

NPs indicated that the NP-WI was easy to use. Acceptability scores were positively rated. Daily data entry took 5 to 7 min to complete. NPs deemed a 4-week collection period sufficient to capture a representative workload sample. The NP-WI captured patient, provider and organizational characteristics and the number of patients seen by NPs.

**Conclusions:**

NP-WI implementation was feasible. The instrument can support healthcare workforce planning with more adequate estimations of NP workload in community-based primary care, and provide greater equity in resource allocation and distribution of NP workload.

## Introduction

Access to primary healthcare services is a global challenge.^1^ Nurse practitioners (NPs) are Master’s-prepared nurses with advanced clinical competencies.^2,3^ NPs autonomously diagnose and treat health conditions in a wide range of community-based practice settings including primary care, long-term care, and home care.^2,3^ NPs improve access to care in these settings.^4^ However, the number of patients seen by NPs is an important consideration.^5^ Wide variability in the number of patients seen by NPs has been noted internationally across primary care-based settings with average numbers ranging from 9 to 15 patients per day.^5^

Workload refers to the amount of time needed to complete an activity,^6^ and caseload is the number of patients under the care of a full-time provider.^5^ Determining an appropriate workload for NPs is complex because of the large number of factors to consider.^7^ Excessive workload and difficulties meeting patients’ needs cause distress for care providers and can increase their intent to leave.^8,9^ Significant challenges have been identified to capture NP workload because patients are often rostered with physicians rather than NPs.^5^ In many jurisdictions, including Canada, most administrative databases do not capture NP activities.^10-12^ In other jurisdictions, such as the United States, some NP care (eg, patients under Medicare) is reimbursed at a reduced rate.^13^ Even though NPs in the United States are required to bill for services using their own unique identifier, such reimbursement practices can encourage organizations to bill under physicians, making NP practice invisible.^12,13^ Direct observations can capture what NPs do.^14,15^ Time and motion studies (*n* = 945 h 25 min) using direct observations were conducted with NPs in 2 provinces in Canada (ie, Québec and Ontario). These studies found statistically significant differences in the time spent by NPs in activities (ie, workload) after taking into account patient (eg, gender, health condition), NP (eg, gender), organizational (eg, practice setting), and system (eg, scope of practice) characteristics.^14,15^ Direct observations represent the gold standard to capture NP activities.^16^ However, they are resource intensive undertakings that are difficult to complete when large numbers of clinicians are involved or if clinicians are located in several regions. In 2024, 1543 NPs were registered in Québec, making it difficult to capture workload across the province.^17^

A recent study conducted in Québec, Canada, by Landry et al^18-20^ aimed to describe the information needs of stakeholders (ie, NPs, physicians, other care providers, decision-makers, patients) for a workload measurement instrument and determine areas of consensus on the elements to include in the tool. Large areas of consensus were found around the types of appointments (eg, face-to-face), number of patients seen per day, and NP years of experience. However, no consensus was reached among participants for the time needed to enter data into the tool, and the number of days needed to accurately represent NP workload and caseload (ie, 2-, 4-weeks). Using these data, a static model was developed as a mock-up of the measure (ie, Nurse Practitioner Workload Index [NP-WI]).

Measuring NP workload is important. Umbrella reviews of indicators sensitive to NP care in community-based primary care settings^4^ and the global perspective of advanced practice nursing^21^ identified critical gaps in research examining NP workload. Weiner et al^22^ and Wang et al^23^ argue that it is crucial to examine feasibility, acceptability and appropriateness of implementation, particularly with new technology. These authors define feasibility as the perception that an innovation can be used successfully. Acceptability represents perceptions that an innovation is agreeable or satisfactory. Appropriateness examines the perceived compatibility of an innovation with the setting or its ability to address a problem. An online workload measurement instrument to collect NP workload data is needed to support workforce planning in Québec and in other jurisdictions internationally.

## Objectives

The overall aim is to determine the feasibility, acceptability and appropriateness of implementing an online workload measure for NPs. More specifically, aim (1) pilot-test an online version of the NP-WI to determine feasibility and appropriateness; and aim (2) explore stakeholder views of the acceptability of using the online tool.

## Conceptual Framework

The Theoretical Framework of Acceptability (TFA)^24^ supported the study. The TFA includes 7 components: (1) *affective attitude* describes how participants feel about an intervention; (2) *burden* represents the perceived effort to participate in the intervention; (3) *ethicality* embodies the fit between an intervention and participants’ values; (4) *perceived effectiveness* represents participants views that the intervention is likely to achieve its goal; (5) *intervention coherence* addresses participants’ understanding of the intervention; (6) *self-efficacy* examines participants’ perceived confidence in their ability to perform required behaviours; and (7) *opportunity costs* incorporate perceptions of benefits (profits, values) that must be given up by participants to engage in the intervention.^24^

## Methods

### Design

A feasibility study was undertaken to implement the online NP-WI.^25,26^ A participatory research approach guided the process and the implementation plan was adapted with participants.^27^ Perspectives of different stakeholders (eg, NPs, patients, decision-makers, physicians) were examined to support long-term sustainability of the implementation.^24,28^ Québec’s healthcare system is primarily publicly-funded and most NPs work as salaried employees of the government. The project was structured around elements known to support successful short team interventions identified following a systematic review.^29^ They include (1) volunteer participation, (2) regular weekly meetings for the first 3 months of the project to quickly identify and address challenges, (3) discussions with participants to share experiences, (4) determination of implementation priorities, and (5) adaptation of scheduled meetings at participants’ request after the first 3 months to meet their needs (minimum once per month). These elements were tested empirically in 2 implementation studies with NPs and other stakeholders in home care and long-term care in Québec, Canada.^10,30,31^

### Instruments

The online NP-WI was developed using activities identified in time and motion studies with NPs in community-based primary care in Québec and Ontario, a qualitative descriptive study to outline information needs of stakeholders and a consensus building exercise using the TRIAGE Method.^14,15,18,32^ The tool, available in English and in French, was launched in January 2024 to establish proof of concept. The instrument includes an NP profile page that is completed once when the NP starts to use the tool, and includes the NP’s years of experience, number of hours and days worked per week. Organizational characteristics include the main and secondary practice settings, presence of an electronic health record (EHR), clerical and nursing supports, presence of other NPs, team size, and level of collaboration in the team. Daily, NPs select the appropriate date and add information to describe the types of appointments and patient characteristics. Time spent in non-clinical role dimensions and NP perceptions of workload (1-very unsatisfied to 4-very satisfied) are indicated daily. Reports for NPs and decision-makers are generated using these data. Decision-makers receive aggregated data for all NPs in their service who enter data in the tool.

The Theoretical Framework of Acceptability (TFA) Questionnaire-Providers^33^ consists of 8 items to assess the domains of acceptability of healthcare innovations described above. The measure takes 2 min to complete. Responses range from 1 to 5 with anchors for each item. Median scores are reported by item. Lower scores for burden and opportunity costs indicate increased acceptability.

Adequacy of Appointment Time Questionnaire-Patients: This 4-item questionnaire takes less than 2 min to complete online or a paper copy. Patients are asked their gender, age (above or below 65 years of age), length of appointment with NP adequate to meet their needs (yes/no), satisfaction with appointment (yes/no).

A semi-structured interview guide explored themes included in the conceptual framework (eg, burden) and perceptions of usability (eg, menus, navigation, content, colours).^34^

### Data Collection

We collected data from January to July 2024. Our target sample size of a minimum of 30 participants for continuous outcomes and 50 participants for binary outcomes was sufficient to capture a range of perspectives and experiences with the NP-WI and consistent with recommendations by Totton et al^35^ and Kunselman^36^ for sample sizes in the context of feasibility studies. The implementation team (*n* = 11) included NPs and decision-makers from the 3 health regions. The team met weekly. They were actively engaged by the research team that included a patient partner (AD) to share experiences and trouble shoot issues (eg, connectivity). The implementation team were asked to share concerns during regular team meetings and via email. Field notes recording meetings, issues, and impressions were circulated weekly to the implementation team to ensure accuracy. The implementation team provided feedback on the NP-WI and supporting material. NPs in each region completed data entry for the online tool. NPs and decision-makers were asked to complete the TFA questionnaire. Patients who were seen by NPs were asked to complete the 4-item questionnaire. Semi-structured interviews were completed with participants to identify different perspectives. Purposive sampling was used to identify participants with different characteristics (professional group, experience, gender, location, favour implementation/not).^37^ For aim 1, in addition to the TFA questionnaire, we measured the number of complete and incomplete entries, and attrition rates to assess feasibility.^38^ Space to write comments was provided in the NP-WI. For aim 2, we explored perceptions of burden when completing the tool (eg, time to enter data, task complexity, technical issues, 2- or 4-week data collection periods), and adequacy of the content of the online training module.^39^

### Data Analysis

For aim 1, descriptive statistics were generated for the TFA questionnaire and NP-WI outputs.

Patient characteristics include patients in vulnerable situations (yes/no), patient’s age <80; patient’s age <70; several people in the exam room (eg, patients needing a translator, family members present for the health visit), number of issues addressed (1 to ≥4), and patients without specific health needs. NP characteristics include NP experience (years), NP gender (male/female), NP perception of workload (1-very unsatisfying to 4-very satisfying), NP full-time work status (yes/no). Full-time status was determined by examining the number of hours worked per week and the number of days worked per week in the NP-WI. The NP level of experience was calculated by subtracting the total duration of leaves for illness (months), maternity/paternity or other work stoppages (months) from the NP years of experience. The values are categorized by the NP-WI as novice (<1 year), beginner (1 to <2 years), competent (2 to <5 years), and expert (≥5 years). Organizational characteristics include the presence of an EHR (yes/no), clerical support (yes/no), nursing support (yes/no), other NPs in the practice (yes/no), team size (small, medium, large), perceived collaboration with healthcare team (1-very dissatisfied to 4-very satisfied), location (urban/non-urban). No additional analyses (eg, inferential statistics) were planned. We did not aim to test specific hypotheses or identify causal mechanisms in this feasibility study.^36,40^ Several authors have advised against this practice in the context of feasibility studies as small sample sizes influence the determination of effect sizes and statistical significance (eg, Arain et al,^41^ Lancaster,^25^ Thabane et al,^42^ Teresi et al^26^). We did not impute values for missing data but monitored non-responses as part of the assessment of feasibility. Pearson et al^43^ argue that to examine an implementation process in the context of a feasibility study, proportions and types of providers are a more adequate measure to answer the research question.

Content analysis was used to analyze the qualitative data.^44^ We remained sensitive to NP expressions of power imbalances or concerns about how the data from the NP-WI would be used by decisions-makers as a potential gender difference.^45^ Interviews were transcribed verbatim. A deductive approach was used to code themes consistent with definitions in the framework and an inductive approach identified emerging themes in the data.^46^ Each data source was analyzed separately and integrated at the analysis phase using joint displays.^47,48^ Triangulation of data sources enhanced our understanding of this complex phenomenon.^49^ Results are reported using the Standards for QUality Improvement Reporting Excellence (SQUIRE) 2.0 guidelines (Supplemental Material).^50^

## Results

Participants were located across the 3 health regions. Participants were primarily women (84%), with a mean age of 38.42 years (standard deviation [SD]: 7.26, median: 38.00, range: 24.00-58.00). On average, they were licensed in their role for 10.58 years (SD: 8.99, median: 8.50, range: 0.67-32.00). Almost all participants worked full-time, and had worked with their team an average of 4.12 years (SD: 3.72, median: 3.00, range: 0.33-13.00) (See Table 1).

**Table 1.** Characteristics of Participants Who Completed the TFA Questionnaire (*n* = 47).

Variables	*n*	%	Mean	Standard deviation	Median	Minimum	Maximum
Gender							
Male	7	15.6					
Female	38	84.4					
Age (years)	43		38.42	7.26	38.00	24.00	58.00
Professional group							
Nurse practitioner	43	93.5					
Decision-maker	3	6.5					
Education							
Master’s	44	95.7					
Specialty certificate	2	4.3					
Employment status							
Permanent full-time	45	97.8					
Permanent part-time	1	2.2					
Licensed or registered in your professional role (years)	46		10.58	8.99	8.50	0.67	32.00
Current role in this organization (years)	46		5.19	4.86	3.00	0.42	25.00
Working with this team (years)	46		4.12	3.72	3.00	0.33	13.00

For aim 1, the team co-developed a training module including a 10-min online training video, user guides, frequently asked questions (FAQs) in English and in French. A communication plan was developed with participants to describe the instrument’s purpose, clarify how it would be used and circulated to NPs across the sites to support uptake. Participants reviewed the training material and provided feedback. Adjustments were made weekly or as needed to adapt the training material using the feedback and respond to questions.

NPs (*n* = 66, response rate: 42%) entered data using the NP-WI to pilot-test data entry, visualize fields included in the tool, and generate reports. NPs entered data for the NP-WI for 128 days, with 45 NPs (68%) completing data entry for at least 20 days (range: 1-60 days). The tool captured practice sites in primary care, long-term care and home care.

The percentage of time spent by NPs was primarily in the clinical (84%) dimension, followed by administration/leadership (8%), education (5%), and research (3%). On average, NPs saw 8.3 patients per day across community-based primary care settings (SD: 4.56, median: 8, range: 1-38) (see Table 2). The number of problems/patient concerns addressed per appointment ranged from 1 (67%), 2 (21%), 3 (8%), and 4 or more (4%). First appointments and follow-up for mental health conditions accounted for 10.5% of interventions. Most NPs (92.8%) indicated they were satisfied or very satisfied with the level of collaboration in the healthcare team, and approximately 2 thirds of NPs (65.8%) reported they were satisfied or very satisfied with their workload. NP perceptions of workload, organizational, team and patient characteristics are described in Table 3. Incomplete data entry was less than 1% except for NP perceptions of workload. Participants indicated that the check box was difficult to see which made it easy to pass over the item, particularly if data entry was completed on their portable phone. Patients (*n* = 420, 99.5%) overwhelmingly responded that they were satisfied with appointments times with NPs, and times were adequate to meet their needs. Reports were generated overall and for each site in table form or pie chart.

**Table 2.** Mean Number of Patients Seen per Day per NP, by Setting.

Settings	Data collection days (*n*)	Number of patients seen per day per NP				
		Mean	Standard deviation	Median	Minimum	Maximum
LTC	17	4.3	2.56	4	1	8
Home care	8	1.88	0.83	2	1	3
Ambulatory care	118	8.78	4.29	8	1	38
Mental health	37	5.42	2.45	5	1	15
Total	128	8.3	4.56	8	1	38

Abbreviations: LTC, long-term care; NP, nurse practitioner.

**Table 3.** Description of NP Perceptions of Workload, Organizational, Team, and Patient Characteristics in the NP-WI.

Variables	Site 1		Site 2		Site 3		Total	
	*n*	%	*n*	%	*n*	%	*n*	%
NP perception of workload								
Satisfied	1782	41.8	2406	60.4	1544	59.4	5732	52.8
Very satisfied	505	11.9	636	16.0	247	9.5	1388	12.8
Dissatisfied	516	12.1	253	6.4	506	19.4	1275	11.7
Very dissatisfied	92	2.2	12	0.3	81	3.1	185	1.7
Missing	1366	32.1	675	17.0	235	9.0	2276	21.0
Setting								
Family medicine group	3337	78.3	2678	67.3	1189	45.5	7204	66.4
Local community service centre	235	5.5	357	9.0	842	32.2	1434	13.2
University-affiliated family medicine group	192	4.5	301	7.6	486	18.6	979	9.0
Medical clinic	207	4.9	445	11.2	0	0.0	652	6.0
Cooperative clinic	258	6.1	133	3.3	0	0.0	391	3.6
Residential and long-term care	0	0.0	41	1.0	75	2.9	116	1.1
Missing	32	0.8	27	0.7	21	0.8	80	0.7
Electronic health record (EHR)								
Yes	4229	99.2	3277	82.3	2471	94.6	9977	91.9
No	0	0.0	678	17.0	121	4.6	799	7.4
Missing	32	0.8	27	0.7	21	0.8	80	0.7
Clerical support								
Yes	4178	98.1	3460	86.9	2342	89.6	9980	91.9
No	51	1.2	495	12.4	250	9.6	796	7.3
Missing	32	0.8	27	0.7	21	0.8	80	0.7
Nursing support								
Yes	3953	92.8	2823	70.9	2218	84.9	8994	82.8
No	276	6.5	1132	28.4	374	14.3	1782	16.4
Missing	32	0.8	27	0.7	21	0.8	80	0.7
Other NP(s) in your practice setting								
Yes	2479	58.2	3123	78.4	2471	94.6	8073	74.4
No	1750	41.1	832	20.9	121	4.6	2703	24.9
Missing	32	0.8	27	0.7	21	0.8	80	0.7
Team size								
Small (5 and less)	302	7.1	983	24.7	103	3.9	1388	12.8
Medium (5-9)	885	20.8	1234	31.0	908	34.7	3027	27.9
Large (more then 10)	3042	71.4	1738	43.6	1581	60.5	6361	58.6
Missing	32	0.8	27	0.7	21	0.8	80	0.7
Level of collaboration								
Very satisfied	2332	54.7	1264	31.7	1681	64.3	5277	48.6
Satisfied	1897	44.5	2436	61.2	469	17.9	4802	44.2
Very dissatisfied	0	0.0	122	3.1	240	9.2	362	3.3
Dissatisfied	0	0.0	133	3.3	202	7.7	335	3.1
Missing	32	0.8	27	0.7	21	0.8	80	0.7
Appointment format								
In-person	2769	65.0	2815	70.7	2125	81.3	7709	71.0
Telephone	1206	28.3	1031	25.9	438	16.8	2675	24.6
Virtual	254	6.0	109	2.7	29	1.1	392	3.6
Missing	32	0.8	27	0.7	21	0.8	80	0.7
Appointment type								
Walk-in or advanced access	1795	42.1	2174	54.6	954	36.5	4923	45.3
Follow-up for chronic illness and/or comorbidities	872	20.5	1047	26.3	752	28.8	2671	24.6
Follow-up for mental health issue	638	15.0	200	5.0	195	7.5	1033	9.5
Follow-up for pregnancy	356	8.4	57	1.4	92	3.5	505	4.7
Periodic exam in children	133	3.1	198	5.0	161	6.2	492	4.5
Rostering/ registering a new patient	132	3.1	51	1.3	113	4.3	296	2.7
Reproductive and sexual health (eg, IUD insertion)	58	1.4	87	2.2	140	5.4	285	2.6
1st appointment for patient with chronic condition	85	2.0	40	1.0	41	1.6	166	1.5
1st appointment for mental health	60	1.4	22	0.6	23	0.9	105	1.0
STBBI appointment	50	1.2	46	1.2	15	0.6	111	1.0
1st appointment for pregnancy	49	1.1	12	0.3	27	1.0	88	0.8
LTC consultation	0	0.0	9	0.2	77	2.9	86	0.8
Home care consultation	1	0.02	12	0.3	2	0.1	15	0.1
Missing	32	0.8	27	0.7	21	0.8	80	0.7
Patients								
Patients in vulnerable situations	977	21.2	404	9.6	550	18.9	1931	16.5
Patients aged 70 years or older	465	10.1	281	6.7	311	10.7	1057	9.0
Patients aged 80 years or older	182	3.9	295	7.0	156	5.4	633	5.4
Patients requiring a translator	45	1.0	62	1.5	45	1.5	152	1.3
Several people in the health visit (excluding well-baby appointment)	80	1.7	64	1.5	82	2.8	226	1.9
Clientele without specific health needs	64	1.4	23	0.5	15	0.5	102	0.9
Homeless clientele	54	1.2	5	0.1	0	0.0	59	0.5

Abbreviations: IUD, intra-uterine device; LTC, long-term care; NP, nurse practitioner; STBBI, sexually transmitted and blood-borne infection.

During the weekly implementation meetings, NPs and decision-makers noted that the number of problems/concerns that were addressed during the patient’s health visit was an important indicator of NP workload. It could be used to help clarify what makes NP practice distinct from other providers (eg, physicians). After noting the high percentage of patients who were seen with 4 or more problems in her caseload, a participant reflected: “*Now I know why I feel overwhelmed!*”

**Figure 1.**
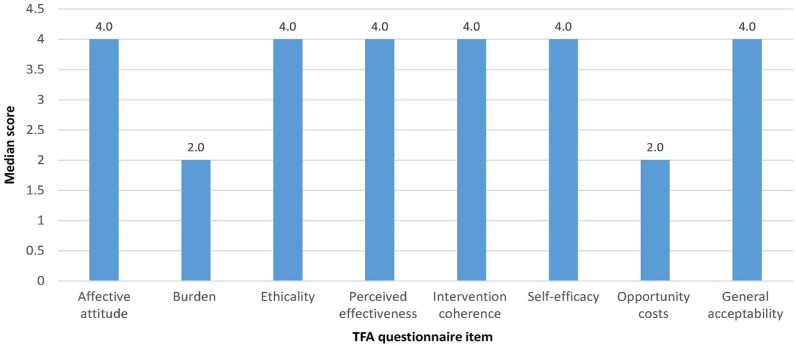
Median scores of acceptability (*n* = 47). Item score ranges and corresponding anchors: Affective attitude (1. very uncomfortable—5. very comfortable), burden (1. none—5. huge), ethicality (1. very unfair—5. very fair), perceived effectiveness (1. strongly disagree—5. strongly agree), intervention coherence (1. strongly disagree—5. strongly agree), self-efficacy (1. very unconfident—5. very confident), opportunity costs (1. strongly disagree—5. strongly agree), general acceptability (1. completely unacceptable—5. completely acceptable).

For aim 2, NPs and decision-makers found that the NP-WI was easy to use and included all the essential elements to measure NP workload. As indicated in Figure 1, the acceptability scores were positively rated with low scores noted for burden and opportunity costs. In the interviews, the NPs noted that data entry took 5 to 7 min each day and this was acceptable to them. They determined that a 4-week data collection period of NP activities was sufficient to capture a representative sample of NP workload. Two weeks were deemed insufficient to capture regular activities that occur less frequently (eg, team meetings). One NP noted: “*The tool is easy to use!*”

Adjustments to the NP-WI included separating appointment types for walk-ins and advanced access, adding categories for appointment types related to follow-up of test results, completing forms, definitions to the user guide for patients in vulnerable situations, and adding white space around the item for NP perceptions of workload.

## Discussion

A feasibility study was conducted in 3 health regions to implement an online NP-WI. NPs indicated that the NP-WI was easy to use. It took 5 to 7 min per day to complete data entry. The NP-WI was able to account for a wide range of patient, provider and organizational characteristics across community-based primary care settings, the time spent in clinical and non-clinical role dimensions, and the number of patients seen by NPs. Identifying the number of issues or problems addressed per visit may help to distinguish NP practice from other provider roles in healthcare teams. NPs determined that a 4-week collection period was sufficient to capture a representative sample of their workload. The study generated new knowledge to facilitate the uptake of an important health service innovation and identify areas for improvement prior to a wider roll-out.

There remains a paucity of research focussed on nurses and NP workload in primary care.^51,52^ To address these concerns and ensure transparency in the use of the information, NPs were asked to assess their workload directly in the instrument. All participants assessed the adequacy of the instrument’s outputs. Subjective assessments of workload that incorporate professional judgement have been found to enhance appropriate resource allocation to meet patient needs.^53^ The NP-WI can be used to support reflective practice for NPs. Decision-makers can more easily identify unevenly distributed workload. Professional development needs for NPs can also be pinpointed depending on the level of NP experience, changes in NP satisfaction of workload and changes in characteristics of patients seen by NPs (eg, increase in number of homeless persons). To support transparency, NPs and decision-makers have access to reports. These data can be used to promote discussions between NPs and decision-makers about their caseload to ensure that NP roles are used optimally, and to build relationships to improve staffing decisions.^54^ The instrument provides NPs and decision-makers with information to improve NP workforce planning, account for the needs of patients with health issues (eg, home care, mental health, walk-in), optimize NP role implementation and increase access to care.

High workloads have deleterious effects on NPs and patients.^55^ To determine what constitutes a reasonable workload, nurses cited effective workload management, modernizing processes, time management, communication and prioritization as key considerations.^56^ Advanced practice nurses have expressed the need to go beyond the number of patients seen to measure clinical and non-clinical activities as well as distinguish between nursing and non-nursing activities (eg, clerical tasks).^57^ Concerns about the adequacy of workload measurement were voiced as early as 1970.^58^ To support decision-makers, these systems must capture the growing complexity of patient care and work environments to support decisions related to prospective planning of resources, determining current staffing adequacy or retrospectively reviewing past resource allocations and expenditures.^7,11,58,59^

The use of a participatory approach allowed stakeholders to inform all steps of the implementation. Engaging users in the co-creation of technology can enhance uptake and sustainability.^60^ To ensure that the patient’s voice was heard, our patient partner participated in all phases of the study from protocol development to publication. Our patient partner reviewed and discussed findings and generated recommendations. Also, offering both online and paper copy options for the 4-item patient questionnaire greatly increased the response rate and should be considered when patients are asked to complete a survey. We did not include interviews with patients at this stage of instrument development because we believed that it was premature to present the untested instrument to patients for their feedback.

Poor healthcare workforce planning is costly and leads to systemic inequities in care, particularly for underserved populations.^61,62^ Additional research is needed to address the limitations of our research project. The study was conducted in 1 province in Canada with a small sample size and did not include patient outcomes. Research is needed to spread and scale up the NP-WI in other provinces or countries to capture more diverse NP roles, patient populations, and different care contexts.^63^ These authors argued that scaling looks to expand the impact of an innovation and represents an effective strategy to reduce costs and waste in research. A larger sample size with participants in different jurisdictions will allow for a more in-depth analysis of the relationships between patient, NP, team, organization, and system level characteristics. There is a lack of research examining the link between NP staffing decisions and patient outcomes using validated tools.^5,64^ Also, a comparison between online and observational data is needed to ensure the adequacy of the measure. Somensi et al^65^ identified no significant differences between the measure but found that the online tool was a better reflection of real-time data.

## Conclusions

It is feasible to implement the NP-WI. The measure captured patient, provider and organizational characteristics and the number of patients seen by NPs. The instrument can support health workforce planning with more adequate estimations of NP workload and caseload across community-based primary care settings and provide greater equity in resource allocation and distribution of workload for NPs. Ultimately, it will be possible to link decisions about workload and caseload to outcomes and address a long-standing critical gap in NP workforce planning.

## Supplemental Material

sj-pdf-1-jpc-10.1177_21501319251321302 – Supplemental material for Implementing an Online Instrument to Measure Nurse Practitioner Workload: A Feasibility Study Supplemental material, sj-pdf-1-jpc-10.1177_21501319251321302 for Implementing an Online Instrument to Measure Nurse Practitioner Workload: A Feasibility Study by Kelley Kilpatrick, Véronique Landry, Eric Nguemeleu Tchouaket, André Daigle and Mira Jabbour in Journal of Primary Care & Community Health
