# Supplementary material for: Implementing an Online Instrument to Measure Nurse Practitioner Workload: A Feasibility Study
Source: J Prim Care Community Health. 2025 Feb 19;16:21501319251321302. doi: 10.1177/21501319251321302 (PMC11840855; doi:10.1177/21501319251321302)
Supplement: sj-pdf-1-jpc-10.1177_21501319251321302 – Supplemental material for Implementing an Online Instrument to Measure Nurse Practitioner Workload: A Feasibility Study [file sj-pdf-1-jpc-10.1177_21501319251321302.pdf]

Reporting checklist for quality improvement in health care.

Based on the SQUIRE guidelines.

Instructions to authors

Complete this checklist by entering the page numbers from your manuscript where readers will find each of the items listed below.

Your article may not currently address all the items on the checklist. Please modify your text to include the missing information. If you are certain that an item does not apply, please write "n/a" and provide a short explanation.

Upload your completed checklist as an extra file when you submit to a journal.

In your methods section, say that you used the SQUIREreporting guidelines, and cite them as:

Ogrinc G, Davies L, Goodman D, Batalden P, Davidoff F, Stevens D. SQUIRE 2.0 (Standards for QUality Improvement Reporting Excellence): revised publication guidelines from a detailed consensus process

		Reporting Item	Page Number
**Title**			
	#1	Indicate that the manuscript concerns an initiative to improve healthcare (broadly defined to include the quality, safety, effectiveness, patientcenteredness, timeliness, cost, efficiency, and equity of healthcare)	1
**Abstract**			
	#02a	Provide adequate information to aid in searching and indexing	1
	#02b	Summarize all key information from various sections of the text using the abstract format of the intended publication or a structured summary such as: background, local problem, methods, interventions, results, conclusions	1
**Introduction**			

Problem description	#3	Nature and significance of the local problem	2
Available knowledge	#4	Summary of what is currently known about the problem, including relevant previous studies	2-3
Rationale	#5	Informal or formal frameworks, models, concepts, and / or theories used to explain the problem, any reasons or assumptions that were used to develop the intervention(s), and reasons why the intervention(s) was expected to work	2-3
Specific aims	#6	Purpose of the project and of this report	3
**Methods**			
Context	#7	Contextual elements considered important at the outset of introducing the intervention(s)	3
Intervention(s)	#08a	Description of the intervention(s) in sufficient detail that others could reproduce it	3-4
Intervention(s)	#08b	Specifics of the team involved in the work	3-4
Study of the Intervention(s)	#09a	Approach chosen for assessing the impact of the intervention(s)	4
Study of the Intervention(s)	#09b	Approach used to establish whether the observed outcomes were due to the intervention(s)	4-5
Measures	#10a	Measures chosen for studying processes and outcomes of the intervention(s), including rationale for choosing them, their operational definitions, and their validity and reliability	3-4
Measures	#10b	Description of the approach to the ongoing assessment of contextual elements that contributed to the success, failure, efficiency, and cost	4
Measures	#10c	Methods employed for assessing completeness and accuracy of data	4
Analysis	#11a	Qualitative and quantitative methods used to draw inferences from the data	4-5
Analysis	#11b	Methods for understanding variation within the data, including the effects of time as a variable	N/A

Ethical considerations	#12	Ethical aspects of implementing and studying the intervention(s) and how they were addressed, including, but not limited to, formal ethics review and potential conflict(s) of interest	Title page
**Results**			
	#13a	Initial steps of the intervention(s) and their evolution over time (e.g., time-line diagram, flow chart, or table), including modifications made to the intervention during the project	6-7, 11
	#13b	Details of the process measures and outcome	6-7, 11
	#13c	Contextual elements that interacted with the intervention(s)	N/A
	#13d	Observed associations between outcomes, interventions, and relevant contextual elements	6-11
	#13e	Unintended consequences such as unexpected benefits, problems, failures, or costs associated with the intervention(s).	11
	#13f	Details about missing data	6
**Discussion**			
Summary	#14a	Key findings, including relevance to the rationale and specific aims	11
Summary	#14b	Particular strengths of the project	11-12
Interpretation	#15a	Nature of the association between the intervention(s) and the outcomes	11-12
Interpretation	#15b	Comparison of results with findings from other publications	11-12
Interpretation	#15c	Impact of the project on people and systems	12
Interpretation	#15d	Reasons for any differences between observed and anticipated outcomes, including the influence of context	11-13
Interpretation	#15e	Costs and strategic trade-offs, including opportunity costs	11-13
Limitations	#16a	Limits to the generalizability of the work	12-13
Limitations	#16b	Factors that might have limited internal validity such as confounding, bias, or imprecision in the design, methods, measurement, or analysis	11-13

Limitations	#16c	Efforts made to minimize and adjust for limitations	11-12
Conclusion	#17a	Usefulness of the work	13
Conclusion	#17b	Sustainability	N/A
Conclusion	#17c	Potential for spread to other contexts	13
Conclusion	#17d	Implications for practice and for further study in the field	13
Conclusion	#17e	Suggested next steps	13
**Other information**			
Funding	#18	Sources of funding that supported this work. Role, if any, of the funding organization in the design, implementation, interpretation, and reporting	Title page

None The SQUIRE 2.0 checklist is distributed under the terms of the Creative Commons Attribution License CC BY-NC 4.0. This checklist can be completed online using https://www.goodreports.org/, a tool made by the EQUATOR Network in collaboration with Penelope.ai
